# Assessment of resolution and noise in magnetic resonance images reconstructed by data driven approaches

**DOI:** 10.1016/j.zemedi.2023.08.007

**Published:** 2023-09-09

**Authors:** Jonas Kleineisel, Katja Lauer, Alfio Borzì, Thorsten A. Bley, Herbert Köstler, Tobias Wech

**Affiliations:** a Department of Diagnostic and Interventional Radiology, University Hospital Würzburg, Würzburg, Germany; b Comprehensive Heart Failure Center, University Hospital Würzburg, Würzburg, Germany; c Chair of Mathematics IX: Scientific Computing, Institute of Mathematics, University of Würzburg, Würzburg, Germany

**Keywords:** Magnetic resonance imaging (MRI), Machine learning, Convolutional neural network, Local point-spread function, Resolution, g-Factor

## Abstract

**Introduction:**

Deep learning-based approaches are increasingly being used for the reconstruction of accelerated MRI scans. However, presented analyses are frequently lacking in-detail evaluation of basal measures like resolution or signal-to-noise ratio. To help closing this gap, spatially resolved maps of image resolution and noise enhancement (g-factor) are determined and assessed for typical model- and data-driven MR reconstruction methods in this paper.

**Methods:**

MR data from a routine brain scan of a patient were undersampled in retrospect at R = 4 and reconstructed using two data-driven (variational network (VN), U-Net) and two model based reconstructions methods (GRAPPA, TV-constrained compressed sensing). Local resolution was estimated by the width of the main-lobe of a local point-spread function, which was determined for every single pixel by reconstructing images with an additional small perturbation. G-factor maps were determined using a multiple replica method.

**Results:**

GRAPPA showed good spatial resolution, but increased g-factors (1.43–1.84, 75% quartile) over all other methods. The images delivered from compressed sensing suffered most from low local resolution, in particular in homogeneous areas of the image. VN and U-Net show similar resolution with mostly moderate local blurring, slightly better for U-Net. For all methods except GRAPPA the resolution as well as the g-factors depend on the anatomy and the direction of undersampling.

**Conclusion:**

Objective image quality parameters, local resolution and g-factors have been determined. The examined data driven methods show less local blurring than compressed sensing. The noise enhancement for reconstructions using CS, VN and U-Net is elevated at anatomical contours but is drastically reduced with respect to GRAPPA. Overall, the applied framework provides the possibility for more detailed analysis of novel reconstruction approaches incorporating non-linear and non-stationary transformations.

## 1 Introduction

Together with recent advances in machine learning and neural network-based computer vision, data-driven methods have seen increasing interest in the MRI academic literature. Several distinct tasks are suited for the application of machine learning, including reconstruction of undersampled data [1], [2]. The data-driven modelling capabilities of neural networks have shown enormous potential for improving and accelerating reconstruction tasks, and are capable of coping with undersampled acquisitions.

However, unlike in classical MRI, the transformation properties and quality of advanced reconstruction methods, data-driven as well as model-driven, depend on local structure and are not uniform across the imaged field of view. Therefore signal abnormalities, e.g. pathological structures, should be judged in view of the local quality and reliability of the imaging method. To this end, local and objective measures of the quality of reconstructions obtained from data-driven methods are needed. These results could moreover prove valuable in the development of new reconstruction methods: Knowing where and how the quality of an image is compromised is certainly crucial for precisely analyzing and systematically improving a reconstruction technique.

While “image quality” actually describes a number of several aspects, the performance of new approaches is frequently assessed by determining only a single global score like RMSE or SSIM. This undoubtedly leads to straightforward assessments, however, it also over-simplifies the evaluation in many cases. Especially image resolution is not considered precisely by these scores. Since classical MRI constitutes a linear and shift-invariant (also called stationary) transform in good approximation, the resolution is uniform and can be analyzed by determining a single point-spread function [3]. Model- and data-driven reconstruction methods, however, in most cases introduce non-linear and non-stationary aspects.

To overcome this problem, resolution has to be determined locally, at every spatial location. Suitable techniques have been developed many years ago. Comprehensive theoretical considerations have first been presented by Fessler and Rogers [4]. These were geared towards applications in emission and transmission tomography, where more research was done subsequently [5]. Later, Wech et al. [6] applied these concepts to cardiac MRI, by determining resolution maps for images reconstructed with iterative thresholding compressed sensing algorithms. However, the recent rise of Deep Learning-based, highly non-linear image reconstruction methods makes this topic more current than ever and leaves their assessment with these tools to be desired. Chan et al. [7] lately assessed resolution for more recent reconstruction methods. They showed perturbation responses in single pixels and checkerboard tests for the whole image. This provided an improved assessment with respect to mere visual inspection, however, the proposed maps are not easily interpretable as local resolution was not quantified. Additionally, the only Deep Learning method they evaluated was a direct U-Net reconstruction, while unrolled gradient schemes like the Variational Network architecture proved to provide superior performance in the meantime.

Besides resolution, the signal-to-noise-ratio (SNR) of an MR image is a decisive quality measure. For determining the noise attenuation and amplification properties of reconstruction methods, g-factor maps are well suited. These can be computed by using a multiple-replica method [8]. By combining the evaluation of resolution with that of noise behavior, we aim to obtain a more comprehensive picture of the properties of current MRI reconstruction methods than previous studies.

By investigating resolution and noise of model- and data-driven reconstruction methods, this work has the following aims: First, it serves to better understand methods currently being developed in academic research, thereby closing the gap toward widespread application. Furthermore, it demonstrates how quality of advanced reconstruction methods can be evaluated systematically and how these evaluations can be interpreted. Ultimately, this could increase diagnostic quality by aiding the analysis of MR images.

## 2 Methods

### 2.1 Measuring image resolution with local point-spread functions

#### 2.1.1 Theory

Classical MRI can be regarded as a linear and shift-invariant system in good approximation. This allows for elegantly studying its characteristics using point-spread functions [3], [6]. However, both linearity and shift-invariance do not hold true for many modern reconstruction methods.

Let $T:R^{n}\to R^{m}$ denote an operator representing such a reconstruction method, which maps some object O onto a representation by an image I=T(O). Note that the complex case is also covered by choosing *n* and *m* appropriately and using the isomorphy $R^{2}≃C$. If we assume T to be differentiable, then we can characterize the local behavior of T in any given pixel $a\in \left\{1,\cdots ,n\right\}$ of the object O as

$$T\left(O+be_{a}\right)=T\left(O\right)+b\nabla T\left(O\right)e_{a}+r\left(b\right)$$

where it holds $\lim_{b\to 0}\frac{r(b)}{b}\to 0$. The differential of T in O is denoted by $\nabla T\left(O\right)$ and $e_{a}={\left(\delta_{\mathrm{ka}}\right)}_{k=1,\cdots ,n}\in R^{n}$ denotes an object consisting of a single point of magnitude b>0. Thus, the partial derivative ∇T(O)e_a_ contains all information about the local behavior of T in the pixel of O. If b>0 is small enough, it can be approximated by

$$LPSF\left(T,O,a\right):=\frac{\left(T\left(O+be_{a}\right)-T\left(O\right)\right)}{b}\approx \nabla T\left(O\right)e_{a},$$

which we call the *local point-spread function (LPSF)* [4], [6] of T in the pixel a for the object O. It can be thought of as the change of the reconstruction $T\left(O\right)$ at location a, given a small change in the reconstructed object O at location a. It is easy to see that if T is linear, the LPSF does not depend on O, and if it is additionally shift-invariant, it does not depend on a.

The key idea for determining resolution from the shape of an LPSF is that details in the input can be distinguished, if they are represented by distinguishable features in the reconstruction [4], [6]. According to the Rayleigh criterion [9], two objects can be separated if the signal intensity between the two maxima of the point-spread functions drops to below 81% of the maximal value. In the case of fully sampled acquisition with Fourier transform reconstruction, the LPSF is sinc-shaped, and one finds that the width of the main lobe at $\frac{2}{\pi }\approx 64\%$ of the maximal height is the minimal distance that two objects need to have, in order to be perceived as separate. Therefore, we choose to measure resolution by the width of the main lobe of an LPSF at 64% of its maximal height. Any width larger than 1 corresponds to a loss of resolution.

#### 2.1.2 Application to undersampled MRI

**Figure 1:**
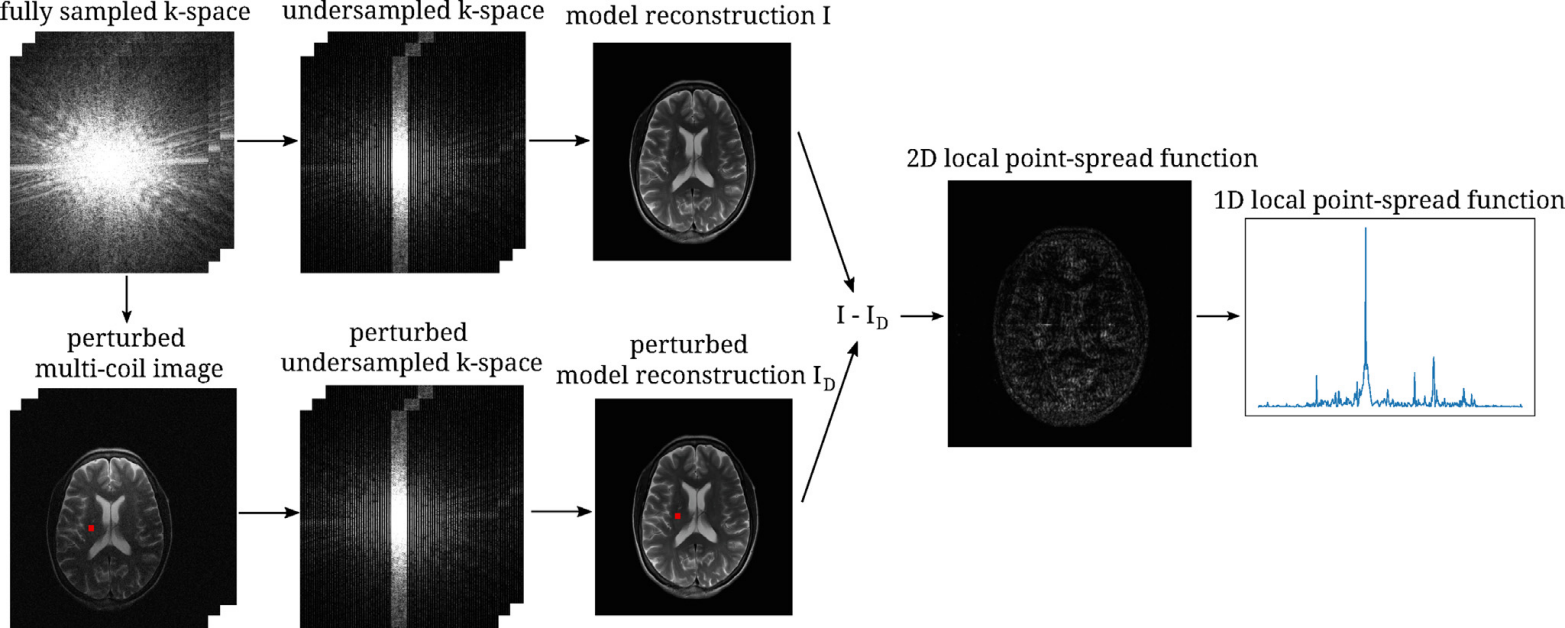
Illustration of the approach for determining local point-spread functions. Fully sampled k-space data is retrospectively undersampled with and without previous perturbation in a single pixel (marked red). By subsequently applying some reconstruction method on the two k-space data, we obtain two model reconstructions. The difference of these represents the 2D local point-spread function (LPSF) for the location of the perturbed pixel. A horizontal or vertical profile is then used for measuring the width of the main lobe.

Now, assume that we have some fully sampled MRI k-space data, which represents some real-world object O, together with a reconstruction method denoted by T. Then we apply the concepts described in the previous section as follows:

First, we transform O coil-wise to image space. For a spatial location a, the image is perturbed by adding a small perturbation to the pixel in a. Empirically, we found that $T\left(O+be_{a}\right)-T\left(O\right)\propto b$ holds true approximately if we choose the amplitude of the perturbation no larger than 0.1% of the maximum of the root-sum-of-squares reconstruction of O for all the tested reconstruction methods and for both of the images that we applied them to. The amplitude of the perturbation in each coil is chosen proportional to the signal level of the coil in a. Furthermore, we found that for the examples studied in this paper, the complex phase of the perturbation had no substantial effect, and thus we always keep the phase of perturbed pixels constant. For the perturbed coil-wise images we then transformed to k-space and applied undersampling. From these “raw data”, we then computed a perturbed reconstruction. An unperturbed reconstruction was obtained simply by applying the model reconstruction to the undersampled original data. As detailed above, the difference between the perturbed and unperturbed reconstruction then yields the LPSF. The entire process is illustrated in Fig. 1.

From the two-dimensional LPSF, the resolution in the two spatial dimensions was then determined from the shape of the LPSF. For this, we extracted the row (respectively, the column), and applied 5-fold Fourier interpolation before measuring the width of the main lobe at 64% of its maximum. This procedure yielded two resolution maps, for the resolution in horizontal and vertical direction.

### 2.2 Determining g-factor maps with a pseudo multiple replica method

Model and data driven reconstructions are typically not shift-invariant. The spatially resolved g-factor quantifies any transformation-immanent noise amplification in addition to the reduced SNR due to reduced scan time. In the following, for determination of g-factor maps we use a pseudo multiple replica method similar to [10].

For some given k-space data, pseudo repetitions of a measurement are simulated by adding synthetic noise. Phased coil arrays exhibit white Gaussian noise, which is correlated due to the coupling of the coils between each other. We thus determined the amplitude and correlation of the noise in the receiver array with a separate acquisition, where no RF-pulses are played out. If $N_{i,k}$ denotes the k’th noise value obtained from the *i*’th coil, then

$$\Psi_{i,j}=\frac{1}{n}\sum_{k=1}^{n}N_{i,k}N_{j,k}^{*},$$

gives the covariance matrix, from which we can obtain appropriate synthetic noise via

$$N_{i,k}^{\mathrm{syn}}=\sum_{j=1}^{c}\Psi_{i,j}^{\frac{1}{2}}N_{j,k}^{\mathrm{std}},$$

where $N_{j,k}^{\mathrm{std}}$ is normally distributed Gaussian noise. We chose to obtain $n_{w}=1000$ pseudo-repetitions of the measurement. These can be reconstructed directly (inverse Fourier transform and coil combination) as well as through some advanced (non-stationary) method after subsampling. This yields $n_{w}$ accelerated and non-accelerated reconstructions, from which the point-wise standard deviations $\sigma_{\mathrm{acc}}\left(x,y\right)$ and $\sigma_{\mathrm{normal}}\left(x,y\right)$ can be computed. The g-factor map is then given by

$$g\left(x,y\right)=\frac{\sigma_{\mathrm{acc}}\left(x,y\right)}{\;\sigma_{\mathrm{normal}}\left(x,y\right)\sqrt{R}},$$

where R denotes the acceleration factor in the subsampling. It is present in the definition of the g-factor, since simply due to a decrease in acquisition time, the noise level is expected to increase by a factor of $\sqrt{R}$. If g>1, then noise is increased by more than what is expected from the shortened acquisition, i.e. the method amplifies noise. Conversely, if g<1 then noise is suppressed.

### 2.3 Reconstruction methods

For gaining insights into their reconstruction properties, we chose a selection of popular model-based and data-driven methods:

**GRAPPA**: As classical parallel imaging baseline, we used a GRAPPA [11] implementation [12].

**Compressed sensing:** A TV-regularized compressed sensing (CS) model, which is implemented in the Berkeley Advanced Reconstruction Toolbox (BART) [13]. The optimization problem is solved with the ADMM optimizer [14]. The parameters (number of iterations and regularization weight) were individually adjusted for the T_1_- and T_2_-weighted image.

**Variational network:** For data-driven reconstructions, we used the publicly available challenge dataset and the baseline models of the 2020 fastMRI challenge [15]. The pre-trained models were provided by fastMRI. They are trained on the fastMRI 2020 multicoil brain challenge dataset, consisting of 4469 T_1_, T_1_ post-contrast, T_2_, and FLAIR acquisitions with additional 1378 for validation. The dataset and the models are described in detail in the accompanying paper [15] and a previous publication [16]. The variational network (VN) model consists of 12 cascades, and the regularization terms are realized by U-Nets with 4 up- and downsampling steps and 18 channels in the first layer. The entire VN model has $2.99\cdot 10^{7}$ trainable parameters.

**U-Net**: Like the VN model, the U-Net model was one of the baseline models of the 2020 fastMRI challenge [15], and was trained on the same dataset. It has the same structure as the regularizers in the VN model, but uses 256 channels in the first layer. It has a total of $4.96\cdot 10^{8}$ trainable parameters.

**Figure 2:**
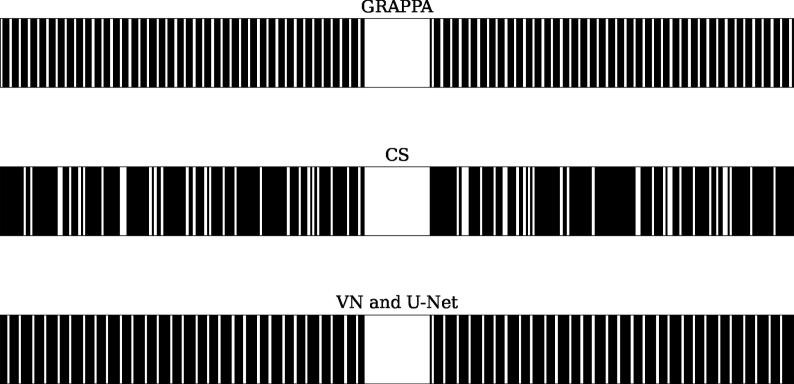
Undersampling patterns with acceleration R = 4 used for masking the T_1_-weighted scans. Only a section of the vertical size is shown. Due to a different matrix size, the random and equispaced masks for the T_2_-weighted scans differ but follow the same characteristics. GRAPPA uses a regular undersampling pattern where every fourth line is sampled and uses the fully sampled center region for kernel calibration (ACS region), but not for reconstruction, such that R = 4 is achieved. The other methods include the ACS region. CS uses a random undersampling pattern, while VN and U-Net alternate between gaps of 4 and 5 missing lines between each sampled lines, to ensure R = 4 and consistency with the masks in the training data.

All methods were applied to data, which were retrospectively undersampled with an acceleration factor of R=4. Undersampling was applied in phase-encoding direction, which corresponds to horizontal direction in image space. The undersampling masks, which differ between the methods due to their specific requirements, can be seen in Fig. 2. For all methods, a fully sampled region in the center of k-space with size 8% of phase encoding lines, called the autocalibration signal (ACS) region, was used. For GRAPPA, the calibration was performed on this region and the reconstruction then applied to k-space where every fourth line was sampled, without ACS region, to ensure R=4. For CS, a random undersampling pattern with ACS region was used. For both VN and U-Net, an undersampling pattern with ACS region was used, where the width of the gaps between the sampled lines alternates between 4 and 5 lines. This matches the undersampling in the training data. Due to a different matrix size, the random and equispaced masks for the T_1_-weighted scans differ, but follow the same principles.

### 2.4 Acquisitions

Raw data of a fully sampled MR-investigation of the brain on a clinical 3T scanner (MAGNETOM Prisma fit, Siemens Healthcare GmbH, Erlangen, Germany) was exported as a representative dataset. The subject was a 57-year old woman. Images were acquired to rule out cerebral manifestation of a B-Non-Hodgkin Lymphoma. Apart from a few unspecific white matter lesions no intracranial pathology was found. Transversal T_1_-weighted images were acquired using a spoiled gradient echo sequence (TE = 4.28 ms, TR = 321 ms, flip angle 90°, FOV = 235 × 235 mm [2], in-plane spatial resolution = 0.61 × 0.68 mm^2^, matrix size 384 × 346) and T_2_-weighted images were acquired using a TSE-sequence (TE = 91 ms, TR = 6610 ms, flip angle 150°, FOV = 235 × 235 mm^2^, in-plane spatial resolution = 0.46 × 0.51 mm^2^, matrix size 512 × 461), well within the distribution of the NYU training dataset. A head coil with 20 channels was used. As described in section 2.2, we additionally acquired a noise measurement for computing g-factors.

Our study was approved by the institutional review board of the University of Würzburg and the requirement of written informed consent by the study participant was waived due to the retrospective study design. The data was fully anonymized for data analysis.

## 3 Results

### 3.1 Qualitative comparison of reconstructions

**Figure 3:**
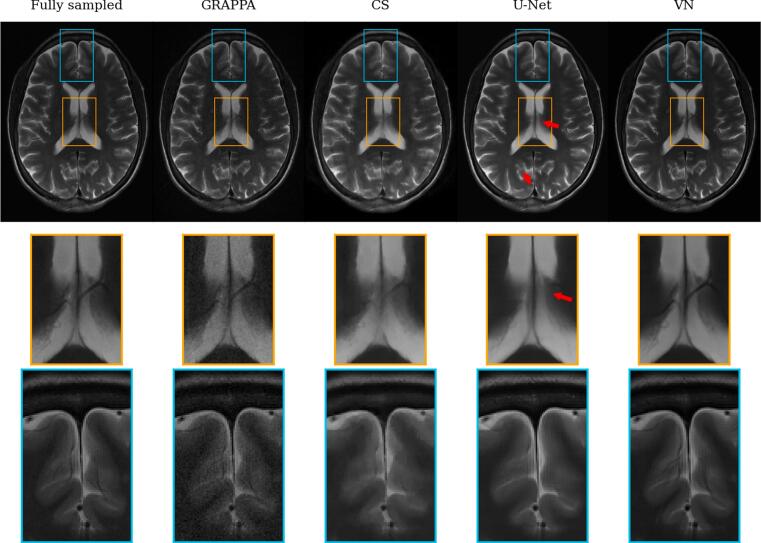
Reconstructions of a central slice of the T_2_-weighted acquisition by the tested methods. The red arrows indicate substantial reconstruction errors in the U-Net output where anatomy is misrepresented due to hallucinations. The yellow and blue boxes show anatomical structure in detail in the lower two rows. One can see substantial noise in the GRAPPA reconstructions, while the other model reconstructions appear smoothed and alter the appearance of small details in some cases.

**Figure 4:**
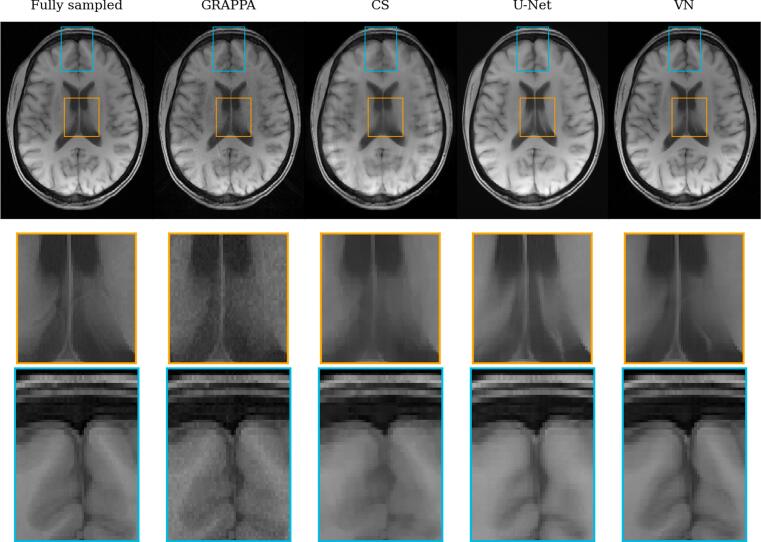
Reconstructions of a central slice of the T_1_-weighted acquisition by the tested reconstruction methods. The yellow and blue boxes show anatomical structure in detail in the lower two rows. Like in the T_2_-weighted images, one can see substantial noise in the GRAPPA reconstructions, while the other model reconstructions appear smoothed and alter the appearance of small details in some cases.

Figure 3, Figure 4 show reconstructions of a central slice of the T_2_- and T_1_-weighted acquisitions by the reconstruction methods as detailed above. In general, all methods seem to correct for undersampling artifacts at R = 4 effectively. However, especially in the enlarged ROIs (orange and blue boxes), some important differences can be noticed: With respect to detail and sharpness, GRAPPA seems to preserve all information present in the reference, but adds some noise. CS exhibits some clearly noticeable spatial blurring, such that finer anatomical details are lost. The U-Net reconstruction appears to exhibit low apparent noise and only minimal blurring. But, critically, in the locations indicated by red arrows in the T_2_-weighted images, anatomy is misrepresented by hallucinations which hide specific features. The VN on the other hand does not show similar systematic errors for this example, and exhibits an apparently high image quality similar to the reference.

### 3.2 Resolution maps

**Figure 5:**
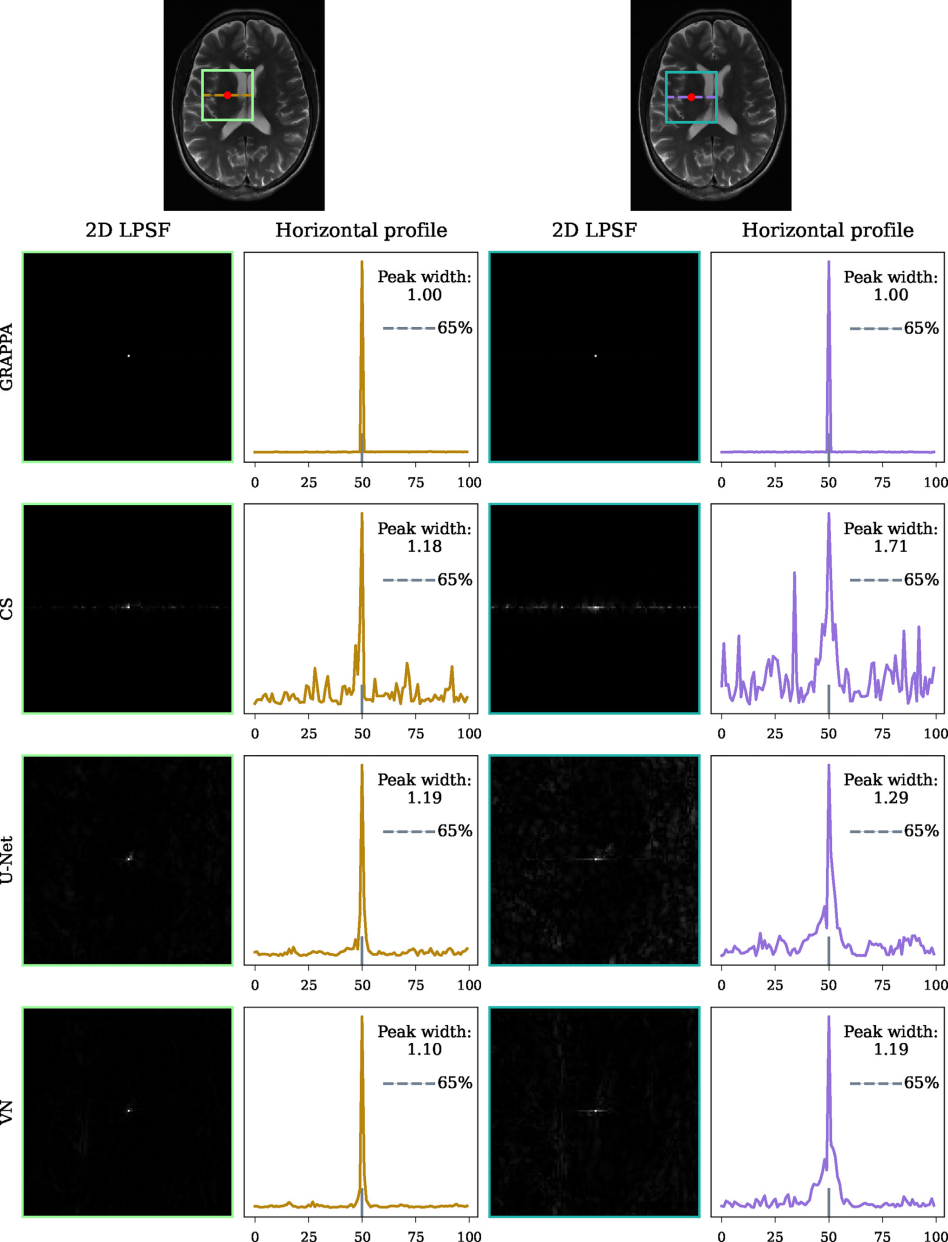
Representative local point-spread functions (LPSF) for two pixels of the T_2_-weighted image for all tested reconstruction methods. The first row shows the respective locations of the pixels and indicates a region of interest (ROI) to be investigated. Below, the 2D LPSFs in this ROI as well as their profile in horizontal direction are shown. The corresponding widths that were computed from these profiles at 65% of the peak height after interpolation are noted. The locations were selected to show an example of a pixel where the methods exhibit narrower (in the two left columns) and wider (in the two right columns) widths of the LPSF’s main lobe, corresponding to lower and higher level of local blurring. Especially in CS and U-Net, one can observe substantial signal in the 1D and 2D LPSF outside the main lobe.

Representative LPSFs, one with a narrow and one with a wider main lobe can be seen in Fig. 5. In the 2D LPSFs, one can see that in particular CS and U-Net also exhibit changes in pixels sometimes spatially quite far away from the location where the perturbation was set.

**Figure 6:**
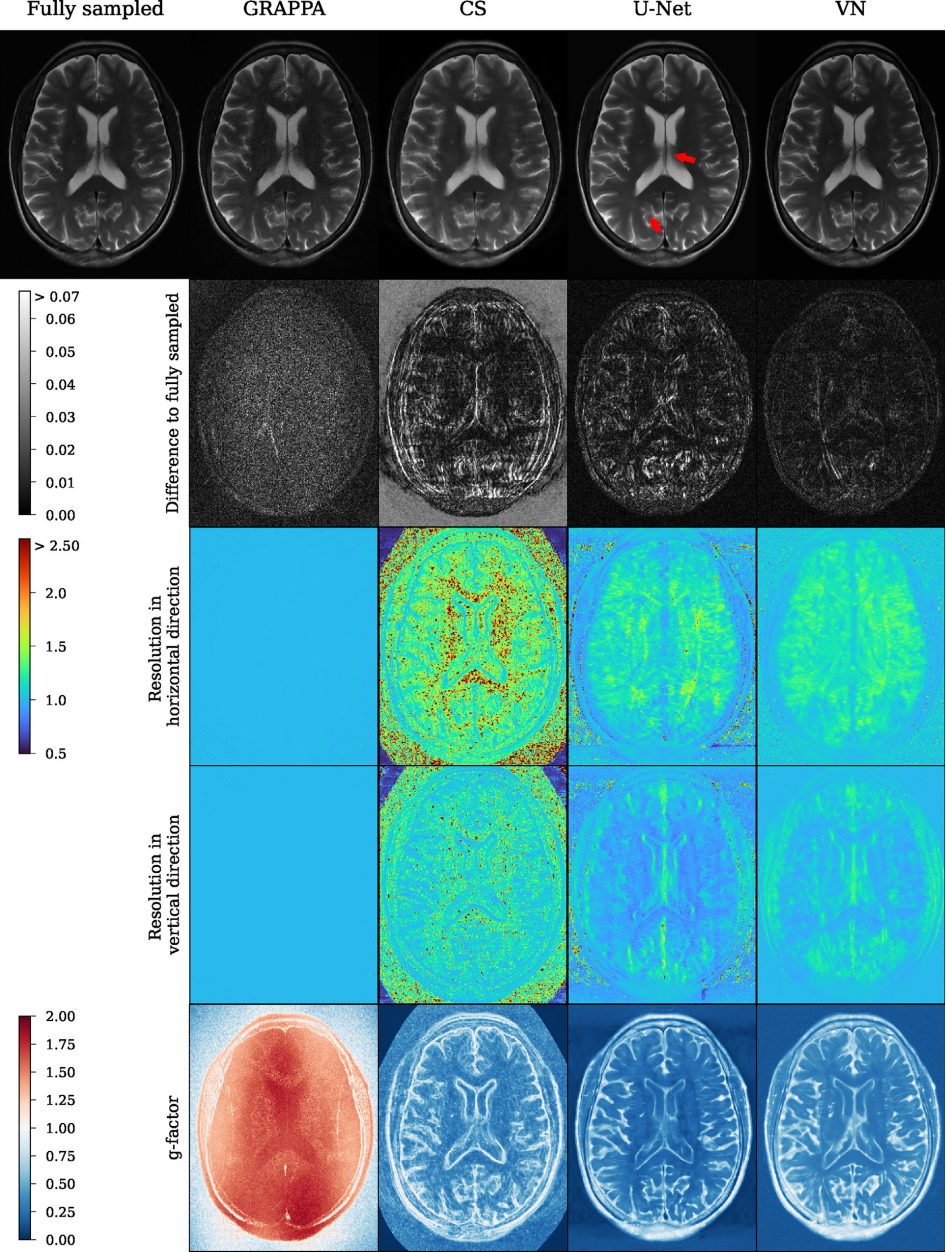
Reconstructions of a T_2_-weighted slice by GRAPPA, compressed sensing (CS), U-Net and variational network (VN), together with error maps, resolution maps in both spatial directions and g-factor maps. The red arrows indicate substantial reconstruction errors in the U-Net output where anatomy is misrepresented due to hallucinations (see also Fig. 3). Since the output of the reconstruction models is scaled arbitrarily, for computing the error maps the output was rescaled with a factor minimizing the l_2_-difference to the reference. The background of the CS reconstruction appears bright in the error map due to the root-sum-of-squares coil combination, which leads to noise with mean > 0 in the background of the reference, while the CS model removes the noise prior to coil combination. The width of the main lobe of the local point-spread function (LPSF) *w* is interpreted as a measure of resolution (3^rd^ and 4^th^ row), where values larger than 1 correspond to a loss of resolution, i.e. local blurring. GRAPPA shows no blurring throughout all locations. In CS and deep learning-based methods, *w* correlates with anatomical structure. Undersampling was applied in horizontal direction (see Fig. 2), where generally more blurring is indicated by the resolution maps. Values of the g-factor below 1 correspond to a suppression of noise, while values above 1 show noise amplification. All methods except GRAPPA suppress noise in most areas, while GRAPPA shows the known characteristics of noise amplification.

**Figure 7:**
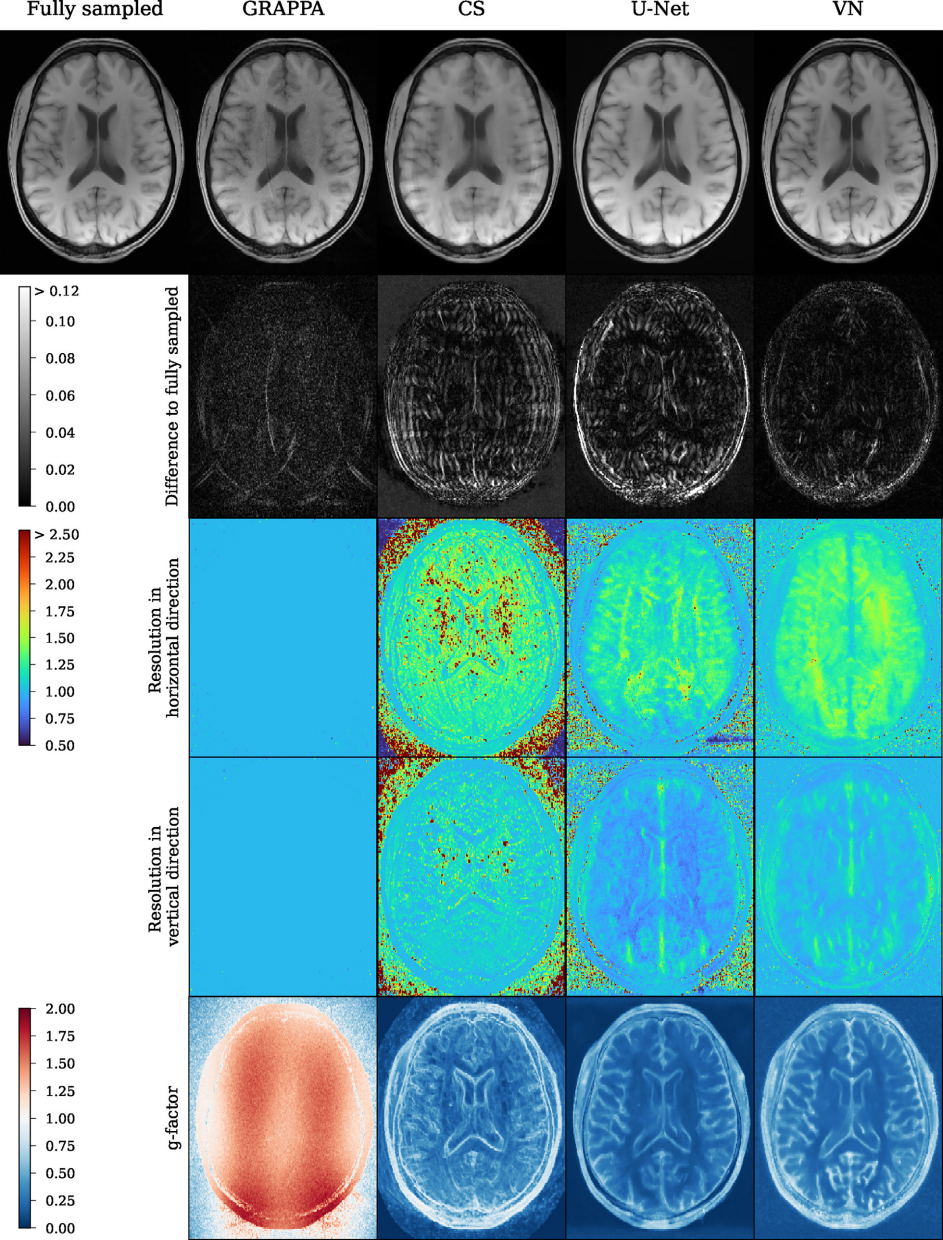
Reconstructions of a T_1_-weighted slice by the tested methods together with resolution maps in both spatial directions and g-factor maps. The results appear similar to the ones obtained in the T_2_-weighted image (Fig. 6), with ideal resolution for GRAPPA and blurring correlated with anatomical structure for the remaining model- and data-driven reconstruction techniques. With respect to noise, GRAPPA again features slightly elevated g-factors while the application of CS, U-Net and VN resulted in denoised images.

**Figure 8:**
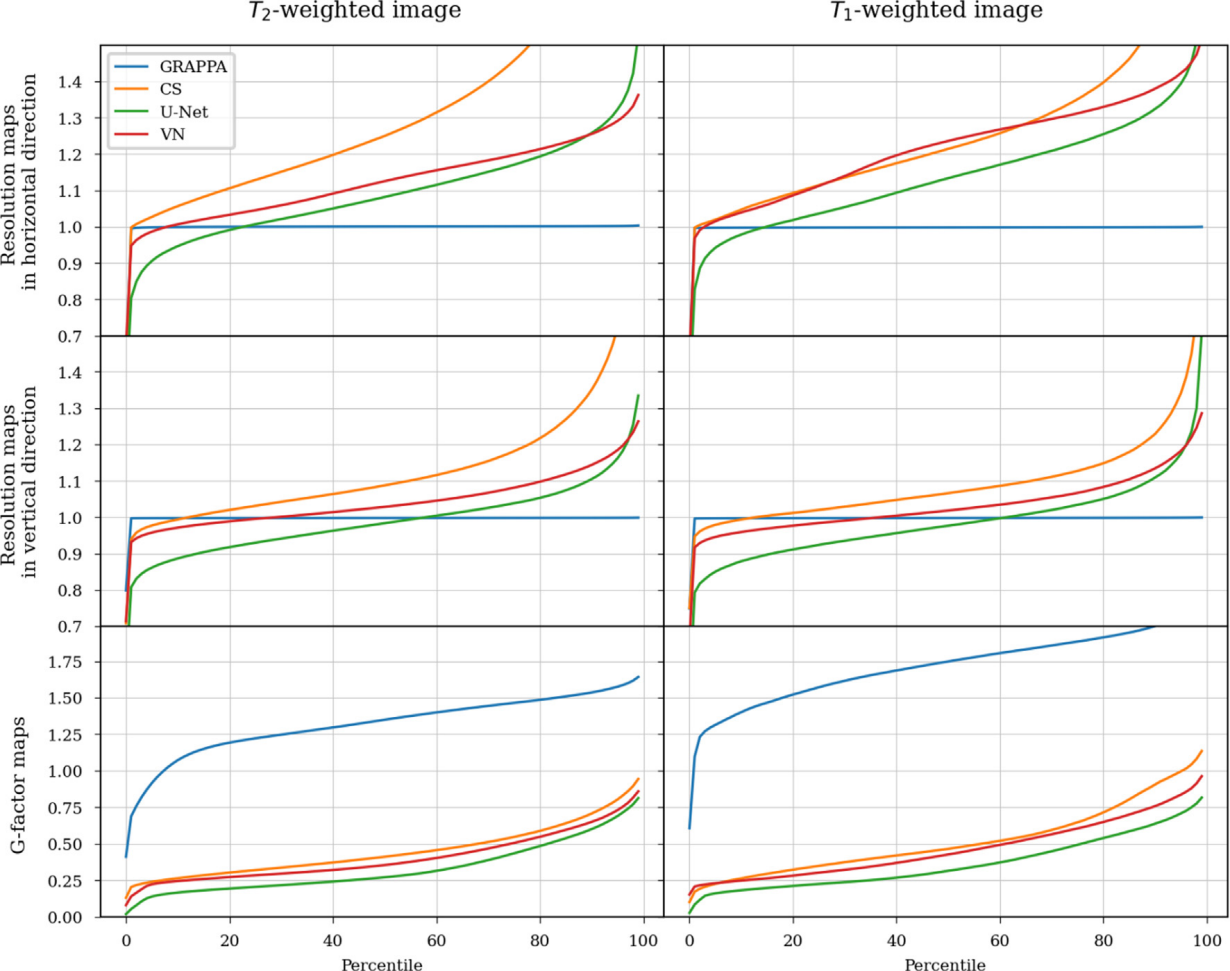
Cumulative frequency analysis (percentile plots) of the resolution maps in horizontal and vertical direction and the g-factor maps that are shown in Figure 6, Figure 7 for the T_2_ and T_1_-weighted image. A mask was applied to only take values inside the anatomy into account. The x-axis specifies percentages, while the y-axis gives the corresponding percentile of the number of pixels in the image. Resolution scores above 1 indicate local blurring in those pixels. The g-factor measures noise amplification (if > 1) or suppression (if < 0). From the curves, it is obvious that GRAPPA shows no blurring basically everywhere, and high g-factors above 1 in most pixels. The other methods exhibit g-factors below 1 almost everywhere. For the resolution scores, CS shows values substantially above 1 in most pixels, and the highest values of all methods. VN and U-Net appear similar, with the widths of the LPSFs for VN somewhat larger than for U-Net in most pixels. However, from the crossings of the green and red line one can see that U-Net has more outliers.

Figure 6, Figure 7 show the resolution maps in both spatial dimensions obtained from the proposed method for the T_1_- and T_2_-weighted images. Cumulative frequency analysis (CFA) of the resolution maps and g-factor maps is shown in Fig. 8, where a mask was applied to only take pixels in the anatomy into account.

For the GRAPPA method, one can see from the maps and the CFA, that except for some outliers, the maps are constant at a value around 1, indicating no substantial loss in resolution with respect to nominal $k_{\max}$, homogeneously across the image.

Compressed sensing clearly exhibits the largest broadenings of the LPSF’s main lobes w, i.e. the lowest resolution. The resolution maps appear non-continuous, with even neighboring pixels being assigned clearly different resolutions. In pixels near edges, the width w of the LPSF’s main lobe is lower than in homogeneous areas, where values between w=1.5-2.5 are common, indicating blurring there. The behavior is similar in the resolution maps for both directions, with more blurring in the undersampled direction.

Similarly, U-Net and VN show blurring in the undersampled horizontal direction in most of the pixels. Their general level of blurring is less pronounced compared to CS and lowest for U-Net. A higher resolution close to 1 is preserved close to edges. In the fully sampled vertical direction, the behavior is reversed, as U-Net and VN show moderate blurring at sharp anatomical edges, while the width of the main lobe is reduced to values smaller than 1 for homogeneous regions in the brain and the ventricles. The maps and in particular the CFA present the resolution of U-Net and VN to be similar, while VN shows slightly higher overall blurring and U-Net exhibits more outliers.

As clearly visible from the resolution maps and CFA, resolution in all methods is noticeably lower in the horizontal direction, where undersampling was applied.

### 3.3 G-factor maps

Figure 6, Figure 7 show the g-factor maps that were computed by the multiple-replica method as detailed above, while the corresponding cumulative frequency analysis (CFA) can be seen in Fig. 8.

GRAPPA exhibits the classical g-factor maps as shown in various studies [8]. Their geometry is driven by the arrangement of receiver coils.

CS, U-Net and VN, all show a drastic denoising. However, this denoising is not present at edges of the images where the g-factor are close to 1. For CS, U-Net and VN, the g-factor maps appear rather similar.

## 4 Discussion

In this work, local spatial resolution and local noise enhancement is determined for data-driven and model-based reconstructions of undersampled MRI data. Local point-spread functions for each pixel and a multiple-replica approach were applied for quantifying resolution as well as noise amplification in four different reconstruction methods. We found that GRAPPA exhibits a homogeneously uncompromised resolution, but shows considerable noise amplification, as it has been described previously [8]. For all other investigated methods, the resolution depended on image structure. TV-regularized CS suffers from a substantial decrease in resolution in homogeneous areas of the image, but not at edges. To a lesser extent, this holds true for the resolution of U-Net and VN in horizontal direction though also some scattered blurring independent of structure can be seen in the resolution maps. In U-Net and VN in vertical direction, the resolution also depends on anatomical structure and is close to 1 around edges. The blurring for data-driven methods is substantially decreased compared to CS, and slightly higher for VN compared to U-Net. G-factor maps are similar between VN and U-Net, showing noise suppression almost everywhere except around hard edges.

While we are confident that the methods for investigating resolution and noise properties used here provide information on the general behavior of tested reconstruction methods, all results in this paper are initially specific to the anatomy, contrast, acquisition hardware etc. of the images we examined. Besides general similarity, some differences between the T_1_- and the T_2_-weighted image remain, like lower resolution in the T_1_-weighted image in the horizontal direction than in the T_2_-weighted one in all methods, especially the VN (see Figure 6, Figure 7, Figure 8) and visibly lower reconstruction quality in the CS reconstruction of the T_1_-weighted image (see Figure 3, Figure 4) than of the T_2_-weighted one. Since these issues are consistent with the fact that the T_1_-weighted image was acquired (see Section 2.4) with a lower in-plane spatial resolution (0.61 × 0.68 mm^2^) than the T_2_-weighted one (0.46 × 0.51 mm^2^), we think that the acquired resolution and matrix size of the input data does have some effect on the performance of the reconstruction methods and the methods for examining resolution and noise. For data-driven methods, a good match between the training dataset and test images is also important. Investigating these relationships in detail in a more comprehensive study including a variety of anatomies, contrasts, resolutions and hardware would be a worthwhile goal for future work.

We found that there is a substantial difference in the width of the LPSF’s main lobe for the vertical and horizontal directions. The values for *w* in the horizontal direction, where undersampling was performed, were clearly higher than in the vertical direction, see Figure 6, Figure 7, Figure 8. This might be taken into account when choosing the direction in which to perform undersampling.

The general idea of introducing some kind of perturbation to evaluate how a reconstruction method responds has already been used in medical imaging [4], [5], [6], [7]. Nevertheless, the approach of measuring the LPSF’s main lobe has profound advantages over some previously presented methods. In particular, through the process of measuring the width of local point-spread functions, we obtain a *quantitative* measure of resolution, that can easily be interpreted as the ability to discern information by means of a single map. However, this comes at the cost of losing some of the information present in the 2D LPSFs, as only the width of its main lobe is measured and the rest of its shape is not considered. Furthermore, the spatially resolved g-factor also provides clear information on the amplification or suppression of noise for the individual imaging and reconstruction process.

In our results, we saw a clear difference in the maps of resolution and noise between parallel imaging, represented by GRAPPA, and the other reconstruction methods CS, U-Net and VN. GRAPPA interpolation is achieved by a convolution in k-space, separately on the data of each receiver coil. The uniform operation in k-space allows GRAPPA to retain ideal resolution, but at the cost of increased noise. Due to their similarity with GRAPPA in terms of mechanisms and qualitative output, we believe that an assessment of resolution of other parallel imaging techniques like SENSE would have similar results, though a detailed investigation would be of interest. The other examined methods (CS, U-Net and VN) are applied in image space. U-Net operates only on a coil-combined reconstruction. CS and VN use the spatial information by the coil sensitivity maps in their data consistency terms, but not in their regularization terms, which operate on coil-combined images. We consider these mechanisms as a trade-off between resolution and noise, as it allows them to achieve noise suppression and good artifact correction, but results in the introduction of local blurring.

As the underlying goal of MRI is answering clinical questions, it is important how our results translate to the diagnostic value of images reconstructed with the examined methods. For GRAPPA, we did not find any impairment by blurring or otherwise augmented anatomy, in accordance with the theory of the method. The risk here is that the noise is increased to a level where subtle anatomical or pathological details would get lost. The other methods, on the contrary, consistently reduced noise. However, all, and CS the most, showed varying degrees of local blurring, which can hide abnormalities. For the data-driven methods U-Net and VN, pathologies that were not represented in the training set pose a further important challenge. Unlike the purely model-based CS method, these data-driven methods have the ability to systematically suppress some image components, or fabricate an output which appears realistic to human observers, while not being representative of the measured data. In general, the reconstruction defects in deep learning-based methods are diverse and often subtle [15]. For example, even though the U-Net reconstructions appear sharp and no residual aliasing artifacts are visible, we observed hallucinations. We want to stress the fact that the dataset was by no means specifically selected for this reason. Other studies have reported similar errors [15], [17]. Though this is certainly a rare occurrence for well-designed methods, the mere possibility raises the still open question of how these kind of reconstruction faults could be discovered, should they appear in potential clinical application.

The methods we presented here can give a spatially resolved indication of the ability to depict small details and the level of noise. High resolution and low noise are certainly important prerequisites for proper reconstructions, and thus the maps we presented can identify areas in which lower quality is possible. However, locally quantifying fidelity of reconstructions to the measured data in a sufficient manner is a further step, and the results presented here do not allow conclusions in this regard. For example, in Figure 6, Figure 7 we saw overall higher values of *w* in VN than in U-Net, and yet the VN does not exhibit obvious reconstruction defects like U-Net. Some recent publications attempt to measure fidelity by repeated reconstructions of various kind, to identify areas in which the reconstructions are associated with uncertainty [18], [19]. Still, we believe that further development of functional and reliable methods that can provide quantifications of local reconstruction fidelity is desirable, and can have a positive impact on the trustworthiness of machine learning driven MRI.

A central assumption in the local approach, was to use sufficiently small perturbations, in order to be able to use local linearity. This is justified, since the reconstruction operator is usually smooth, and thus locally linear. On the other hand, the perturbation should not be chosen too small, to avoid the perturbation being lost in the noise. By reconstructing and checking the linearity condition, we found that, when we choose the amplitude as 0.1% of the maximal pixel, all the tested reconstruction method behave almost linear for both the T_1_ and the T_2_-weighted image. However, this is highly specific to the reconstruction methods and should be checked for each new method and each new image.

Note that, as described in the methods section, we used different retrospective sampling patterns for the methods. This may introduce slightly different representation of information simply due to the undersampling pattern and not directly the reconstruction method. However, using a single pattern for all methods would have introduced a much more substantial bias, since the methods have different requirements for undersampling mask structure, e.g. GRAPPA requires regular undersampling while compressed sensing benefits from random sampling. Furthermore, due to these patterns being so specific to the reconstruction methods, one could actually regard the sampling as a part of the entire method, which consequently has to be included when comparing to other methods.

An obstacle to overcome for widespread application of local point-spread functions may be computational time. For computing a resolution map, a reconstruction with the method to be tested needs to be computed for each pixel of the input image. Since the computation of the LPSF is not computationally demanding, almost all of the time for computing a resolution map is spent on reconstructions. Therefore the computation times for a resolution map depends heavily on the speed of the reconstruction method. This is manageable for fast reconstruction methods, but problematic for slower ones. Thus, to overcome this issue, the reconstruction method itself can be accelerated through efficient implementations or appropriate hardware, like GPUs. Still, the determination of local resolution may not be suited for routine computation in many images.

A clear advantage of the resolution measurement on the other hand is its simplicity. The algorithm requires in essence only the computation of two reconstructions together with some basic signal processing for determining the width of the main lobe of the LPSF. We provide an implementation on GitHub (https://github.com/expRad/).

## 5 Conclusion

We demonstrated methods for objective quality assessment of non-linear and non-stationary MRI reconstruction methods, namely a multiple-replica method for examining noise and a method for measuring local spatial resolution. These were applied to GRAPPA, compressed sensing, variational network and U-Net reconstructions. This study may serve as an example for the kind of critical and objective analysis of novel reconstruction methods that is necessary for advancing these techniques toward clinical application.

## Data Availability Statement

An exemplary implementation of the described methods is distributed by the authors as open source under the link https://github.com/expRad/dlassessment. The patient data can be made available on request due to privacy/ethical restrictions.

## Declaration of Competing Interest

The authors declare the following financial interests/personal relationships which may be considered as potential competing interests: Our research group receives funding from Siemens Healthcare GmbH, which is, however, not specifically attributed to this project.
